# Breaking the Seizure Cycle: Surgical Outcomes of Medial Temporal Lobectomy in Drug-Resistant Epilepsy

**DOI:** 10.7759/cureus.80130

**Published:** 2025-03-06

**Authors:** Sameh M Salama, Elsayed A Elmor, Hany Zahy, Mohammed R Hammouda, Alaa R Ibrahim, Mostafa A Mostafa

**Affiliations:** 1 Neurosurgery, Al-Azhar University of Medicine, Cairo, EGY

**Keywords:** functional neurosurgery, medial temporal lobectomy, seizure control, stereotactic and functional neurosurgery, surgical epilepsy treatment

## Abstract

Background: Medial temporal lobe (MTL) lesions are among the most common causes of drug-resistant epilepsy. Surgical management typically involves lesion excision, with or without amygdalohippocampectomy. An alternative approach includes selective hippocampal transection to disrupt the epileptogenic circuit without affecting the memory circuit*.*

Objective: To assess epilepsy control after medial temporal lobectomy for management of drug-resistant temporal lobe epileptic patients.

Methods: This is a prospective cohort interventional study of consecutive series of patients coming to Al-Azhar University Hospitals at the time between 2022 and 2024 presented with drug-resistant temporal lobe epilepsy (TLE) and treated with excision of the lesions in the MTL with or without amygdalohippocampectomy. Postoperative seizure control was assessed using the Engel classification scale during follow-up and anti-epileptic drug (AED) withdrawal.

Results: The Engel classification scale was used at follow-up and AED withdrawal to evaluate postoperative seizure control. The results indicated that seizure control improved after medial temporal lobectomy. The final follow-up showed that 15 respondents (75%) reached Engel class I which means they had no seizures and five respondents (25%) had Engel class II, which means they had rare, non-disabling seizures. These results show that medial temporal lobectomy is effective in reducing seizure frequency and improving the quality of life of patients.

Conclusion: A medial temporal lobectomy, alone or in combination with amygdalohippocampectomy, serves as an effective surgical treatment for drug-resistant TLE. Most patients experience both a reduction in seizure frequency and severity while seizure freedom occurs in the majority after the procedure. These results support surgical management as an essential treatment option for medically refractory epilepsy and highlight the importance of identifying patients for early surgical evaluation. More studies with bigger sample sizes along with extended follow-up periods should be performed to improve patient selection criteria and surgical methods that enhance patient outcomes.

## Introduction

The medial temporal lobe (MTL) is one of the utmost brain regions concerned with memory processing as well as in seizure activity. It lies deep inside the temporal lobe and contains between the collateral sulcus and temporal horn several structures of great importance, namely, the hippocampus, parahippocampal gyrus, uncus, dentate gyrus, and amygdala [1]. The hippocampus often centers the discussions of memory and epilepsy, but the entire contribution of the MTL highly concerned the neural circuits for both cognitive functions and seizure generation.

Anatomically, the medial temporal surface constitutes one of the most complicated regions in the human brain, and to make it intelligible, it has been simplified it into three principal divisions: the anterior, middle, and posterior segments [2]. The anterior segment is usually made up of the uncus and entorhinal cortex and contains a medial elevation, the apex of the uncus [3]. The middle segment contains the parahippocampal gyrus, the dentate gyrus, and the fimbria of the fornix, which are closely related to memory encoding and development of epileptic discharges. The posterior part, represented by the posterior parahippocampal and lingual gyri, would then interact with adjacent brain parts to complete significant neural networks controlling spatial navigation and visual memory [3].

MTL abnormalities were regarded among the major causes of drug-resistant epilepsy; mesial temporal sclerosis being the most prevailing pathology within these cases [4]. However, this region is also implicated in other lesions such as focal cortical dysplasia (FCD), vascular malformations like cavernomas, as well as a variety of tumors. Mixed neuronal-glial tumors (MNGTs), such as dysembryoplastic neuroepithelial tumors (DNT) and gangliogliomas, are usually low-grade and tend to present with early-onset seizures, while astrocytomas are more aggressive in their course [5]. In fact, tumors are thought to underlie as much as 25% of the temporal lobe cases of epilepsy and usually start during childhood and adolescence [6].

When epilepsy becomes refractory to medical treatment, surgical intervention is often the best approach. The conventional procedure consists of lesion removal with or without amygdalohippocampectomy, all with an aim to remove the seizure focus while preserving cognitive functions [7]. In some cases, less radical procedures, e.g., hippocampal transection, may suffice to interrupt the seizure without more serious consequences for memory [8].

## Materials and methods

This prospective cohort interventional study was conducted at Al-Azhar University Hospitals, Cairo, Egypt, between 2022 and 2024. We enrolled a consecutive series of patients who presented with drug-resistant temporal lobe epilepsy (TLE). All participants were managed surgically, with the resection focused on lesions within the MTL. Depending on the clinical and radiological evaluation, some patients underwent lesionectomy alone, while others had additional amygdalohippocampectomy or selective amygdalohippocampectomy.

The group of respondents consisted of patients whose ages ranged from 3 to 50 years and who had the kind of semiology through which epilepsy had been diagnosed as having MTL origin. It was of vital importance to include only the patients whose seizures were uncontrolled by a trial of at least two anti-epileptic drugs (AEDs) or who were intolerant to the side effects of these drugs [9].

Among the exclusion criteria were those patients who were younger than 3 years old or older than 50 years, those with multifocal epilepsy, or with lesions located away from the medial temporal region, and those individuals whose epilepsy were controlled with one or two anti-epileptic medications. Also, patients with comorbid conditions that could interfere with safe surgical intervention were not considered for the study. Ethical approval was received from the Institutional Ethics Committee and the need for informed consent was waived.

The patients underwent a detailed evaluation before the surgical intervention, which included a clinical history along with a neurological examination to confirm seizure semiology. The first thing to do was to have the neuro-imaging tests done. Every single patient was given an MRI of the brain, CT scans, and, if necessary, positron emission tomography (PET) imaging through preoperative work-ups. Equally significant was the role played by the scalp electroencephalography EEG to determine the epileptogenic focus correctly.

Individual surgical management was based on the findings that were made before the surgery. The surgeons used a variety of techniques to carry out the surgery from a simple lesionectomy to the more complex procedures of lesionectomy combined with amygdalohippocampectomy or a selective amygdalohippocampectomy in cases where as little as possible impact was essential to the surrounding functional tissue.

The mean follow-up duration was 1.49 years, with an SD of 0.48 years. During this period, we monitored patients postoperatively and assessed their outcomes primarily using the Engel Epilepsy Surgery Outcome Scale, which provides us with a standardized evaluation of seizure control. The Engel classification system categorizes post-surgical seizure outcomes into four classes. Class I indicates that the patient is seizure-free or experiences only a few early, non-disabling seizures, or seizures occurring solely due to drug withdrawal. Class II signifies that disabling seizures occur rarely over at least two years, though they may have been more frequent immediately after surgery, and nocturnal seizures may still be present. Class III represents worthwhile improvement, where seizure reduction is achieved for prolonged periods but does not exceed two years. Class IV reflects no worthwhile improvement, where seizure reduction is minimal, absent, or worsening may occur.

Aside from seizure outcomes, neuropsychological assessment was conducted and surgical complications were recorded. The data collected were coded carefully and entered into the IBM SPSS software (version 23) for statistical analysis. Quantitative variables were reported either as mean with SD or as median with interquartile range (IQR), depending on their distribution, while qualitative variables were expressed as frequencies and percentages. For categorical data, the Chi-square test was applied; for non-parametric continuous data, the Mann-Whitney test. A p-value of less than 0.05 was considered statistically significant.

## Results

Results

A total of 20 patients were included in the study, with 15 patients (75%) in the non-complicated group and five patients (25%) in the complicated group. An initial comparison of demographic and clinical characteristics revealed no statistically significant differences between the two groups regarding age, sex, seizure presentation, duration of epilepsy, or the lateralization of the lesion (Table 1). For example, the median age in the non-complicated group was 16 years (IQR: 11-21) compared to 26 years (IQR: 12-35) in the complicated group (p=0.484), and no significant differences were observed in terms of seizure semiology or side of involvement (all p-values > 0.267).

**Table 1:** Comparison between complicated and non-complicated patients regarding demographic data and characteristics of the studied patients IQR:  Interquartile range; Sig.: Significance; NS: Not significant *: Chi-square test; ≠: Mann-Whitney test P-value > 0.05: Not significant; P-value < 0.05: Significant; P-value < 0.01: Highly significant

		Complications		Test value	P-value	Sig.
		No‎	Yes			
		n=15	n=5			
Age	Median (IQR)	16 (11–21)	26 (12–35)	-0.699≠	0.484	NS
	Range	3.5–50	6–49			
Sex	Females	8 (53.3%)	2 (40.0%)	0.267*	0.606	NS
	Males	7 (46.7%)	3 (60.0%)			
Presentation	Focal with impaired awareness seizures	12 (80.0%)	4 (80.0%)	0.000	1.000	NS
	Rightt focal colonic to bilateral tonic clonic	1 (6.7%)	1 (20.0%)	0.741	0.389	NS
	Epileptic spasm	1 (6.7%)	0 (0.0%)	0.351	0.553	NS
	Left focal fits in the face and upper limb	1 (6.7%)	0 (0.0%)	0.351	0.553	NS
Duration of eplipsy (years)	Median (IQR)	2 (1.5–4)	6 (3–7)	-1.373≠	0.170	NS
	Range	0.5–10	0.5–15			
Side	Right	8 (53.3%)	2 (40.0%)	0.267*	0.606	NS
	Left	7 (46.7%)	3 (60.0%)			

When comparing lesion types between groups, a statistically significant association emerged: patients with glioblastoma multiforme (GBM) were more likely to experience complications, with three patients (60%) of the complicated group having GBM compared to none in the non-complicated group (p=0.001) (Table 2). Other lesion types, including ganglioglioma, pilocytic astrocytoma, mesial temporal sclerosis, and FCD, did not show a significant association with complications.

**Table 2:** Comparison between complicated and non-complicated patients regarding lesion type of the studied patients This table shows that there was statistically significant relation found between GBM with the occurrence of complications with p-value=0.001, while no statistically significant relation found between other lesion type with occurrence of complications. Sig.: Significance; NS: Not significant; HS: Highly significant; GBM: Glioblastoma multiforme; FCD: Focal cortical dysplasia P-value > 0.05: Not significant; P-value < 0.05: Significant; P-value < 0.01: Highly significant

Lesion type	Complications		Chi-square test	P-value	Sig.
	No‎	Yes			
	n=15	n=5			
Ganglioglioma	3 (20.0%)	1 (20.0%)	0.000	1.000	NS
GBM ‎	0 (0.0%)	3 (60.0%)	10.588	0.001	HS
Pilocytic astrocytoma	3 (20.0%)	0 (0.0%)	0.246	0.619	NS
Arachnoid cyst	2 (13.3%)	0 (0.0%)	0.741	0.389	NS
Mesial temporal sclerosis	2 (13.3%)	0 (0.0%)	0.741	0.389	NS
Pleomorphic xanthoastrocytoma	2 (13.3%)	0 (0.0%)	0.741	0.389	NS
Oligodendroglioma	0 (0.0%)	1 (20.0%)	3.158	0.075	NS
Temporal cavernoma	1 (6.7%)	0 (0.0%)	0.351	0.553	NS
Astrocytoma grade 2	1 (6.7%)	0 (0.0%)	0.351	0.553	NS
FCD	1 (6.7%)	0 (0.0%)	0.351	0.553	NS

The surgical approach employed did not differ significantly between groups (Table 3). Techniques such as trans-sylvian, transcortical, sub-temporal, trans-sulcal, and endoscopic fenestration were similarly distributed between patients with and without complications (all p > 0.389).

**Table 3:** Comparison between complicated and non-complicated patients regarding surgical approach of the studied patients Sig.: Significance; NS: Not significant P-value > 0.05: Not significant; P-value < 0.05: Significant; P-value < 0.01: Highly significant

Surgical approach	Complications		Chi-square test	P-value	Sig.
	No‎	Yes			
	n=15	n=5			
Trans-sylvian	5 (33.3%)	2 (40.0%)	0.073	0.787	NS
Transcortical	5 (33.3%)	2 (40.0%)	0.073	0.787	NS
Sub temporal	2 (13.3%)	1 (20.0%)	0.131	0.717	NS
Trans-sulcal (inferior temporal sulcus)	2 (13.3%)	0 (0.0%)	0.741	0.389	NS
Endoscopic fenestration	1 (6.7%)	0 (0.0%)	0.351	0.553	NS

Outcome analysis demonstrated that the presence of postoperative complications had a significant impact on seizure control, as measured by the Engel Epilepsy Surgery Outcome Scale (Table 4). In the uncomplicated group, 13 patients (86.7%) achieved Engel grade 1, compared to only two (40.0%) in the complicated group (p=0.037). Additionally, the mean follow-up duration was significantly longer in the complicated group (2.0 years) compared to the non-complicated group (1.32 years; p=0.003).

**Table 4:** Comparison between complicated and non-complicated patients regarding Engel grade and follow-up duration Sig.: Significance; S: Significant; HS: Highly significant *: Chi-square test; •: Independent t-test P-value > 0.05: Not significant; P-value < 0.05: Significant; P-value < 0.01: Highly significant

		Complications		Test value	P-value	Sig.
		No‎	Yes			
		n=15	n=5			
Engel grade	Grade 1	13 (86.7%)	2 (40.0%)	4.356*	0.037	S
	Grade 2	2 (13.3%)	3 (60.0%)			
Follow up duration (years)	Mean ± SD	1.32 ± 0.44	2.0 ± 0.0	-3.427•	0.003	HS
	Range	1–2	2–2			

An analysis focusing on Engel grade outcomes revealed that sex and the side of the lesion were significantly related to seizure outcomes (Table 5). Notably, all patients with Engel grade 2 outcomes were male (p=0.010) and exhibited left-sided lesions (p=0.010), while other variables such as age, seizure presentation, and duration of epilepsy did not differ significantly between the groups.

**Table 5:** Comparison between patients with Engel grade 1 and grade 2 regarding demographic data and characteristics of the studied patients IQR: Interquartile range; Sig.: Significance; NS: Not significant; S: Significant *: Chi-square test; ≠: Mann-Whitney test P-value > 0.05: Not significant; P-value < 0.05: Significant; P-value < 0.01: Highly significant

		Engel grade		Test value	P-value	Sig.
		Grade 1	Grade 2			
		n=15	n=5			
Age	Median (IQR)	18 (11–35)	16 (12–16)	-0.350≠	0.727	NS
	Range	3.5–50	6–49			
Sex	Females	10 (66.7%)	0 (0.0%)	6.667*	0.010	S
	Males	5 (33.3%)	5 (100.0%)			
Presentation	Focal with impaired awareness seizures	12 (80.0%)	4 (80.0%)	0.000	1.000	NS
	Right focal colonic to bilateral tonic clonic	1 (6.7%)	1 (20.0%)	0.741	0.389	NS
	Epileptic spasm	1 (6.7%)	0 (0.0%)	0.351	0.553	NS
	Left focal fits in the face and upper limb.	1 (6.7%)	0 (0.0%)	0.351	0.553	NS
Duration of epilepsy (years)	Median (IQR)	3 (2–6)	2 (0.5–2)	-1.285≠	0.199	NS
	Range	1–15	0.5–7			
Side	Right	10 (66.7%)	0 (0.0%)	6.667	0.010	S
	Left	5 (33.3%)	5 (100.0%)			

Table 6 compares the distribution of lesion types between patients with Engel grade 1 and Engel grade 2 outcomes, statistical analysis using the Chi-square test revealed no significant differences in lesion type distribution between Engel grade 1 and grade 2 groups (all p-values > 0.05). Specifically, GBM showed a trend toward higher prevalence in Engel grade 2 (n=3, 60.0%) vs. Engel grade 1 (n=0, 0.0%), but this difference did not reach statistical significance (p=0.278). Similarly, oligodendroglioma was observed exclusively in Engel grade 2 (n=1, 20.0%) vs. (n=0, 0.0%) in Engel grade 1, though this finding was also non-significant (p=0.075).

**Table 6:** Comparison between patients with Engel grade 1 and grade 2 regarding lesion type of the studied patients Sig.: Significance; NS: Not significant; GBM: Glioblastoma multiforme; FCD: Focal cortical dysplasia P-value > 0.05: Not significant; P-value < 0.05: Significant; P-value < 0.01: Highly significant

Lesion type	Engel grade		Chi-square test	P-value	Sig.
	Grade 1	Grade 2			
	n=15	n=5			
Ganglioglioma	3 (20.0%)	1 (20.0%)	0.000	1.000	NS
GBM ‎	0 (0.0%)	3 (60.0%)	1.176	0.278	NS
Pilocytic astrocytoma	3 (20.0%)	0 (0.0%)	0.246	0.619	NS
Arachnoid cyst	2 (13.3%)	0 (0.0%)	0.741	0.389	NS
Mesial temporal sclerosis	2 (13.3%)	0 (0.0%)	0.741	0.389	NS
Pleomorphic xanthoastrocytoma	2 (13.3%)	0 (0.0%)	0.741	0.389	NS
Oligodendroglioma	0 (0.0%)	1 (20.0%)	3.158	0.075	NS
Temporal cavernoma	1 (6.7%)	0 (0.0%)	0.351	0.553	NS
Astrocytoma grade 2	1 (6.7%)	0 (0.0%)	0.351	0.553	NS
FCD	1 (6.7%)	0 (0.0%)	0.351	0.553	NS

However, further analysis indicated that when stratified by Engel outcome, the trans-sylvan approach was significantly associated with a worse seizure outcome, as four patients (80%) with Engel grade 2 outcomes had undergone this approach (p=0.014) (Table 7).

**Table 7:** Comparison between patients with Engel grade 1 and grade 2 regarding surgical approach of the studied patients Sig.: Significance; NS: Not significant P-value > 0.05: Not significant; P-value < 0.05: Significant; P-value < 0.01: Highly significant

Surgical approach	Engel grade		Chi-square test	P-value	Sig.
	Grade 1	Grade 2			
	n=15	n=5			
Trans-sylvian	3 (20.0%)	4 (80.0%)	5.934	0.014	S
Transcortical	6 (40.0%)	1 (20.0%)	0.659	0.416	NS
Sub temporal	3 (20.0%)	0 (0.0%)	0.246	0.619	NS
Trans-sulcal (inferior temporal sulcus)	2 (13.3%)	0 (0.0%)	2.143	0.143	NS
Endoscopic fensteration	1 (6.7%)	0 (0.0%)	0.351	0.553	NS

Table 8 further highlights that residual lesion presence was significantly associated with complications (p=0.009), reinforcing the importance of complete lesion resection in achieving optimal outcomes. In contrast, no significant differences were noted with respect to the overall follow-up duration between Engel grade groups.

**Table 8:** Comparison between patients with Engel grade 1 and grade 2 regarding percentage of patients with postoperative complications, types of complications and follow-up duration of the studied patients Sig.: Significance; NS: Not significant; S: Significant; HS: Highly significant; AED: Anti-epileptic drug *: Chi-square test; •: Independent t-test P-value > 0.05: Not significant; P-value < 0.05: Significant; P-value < 0.01: Highly significant

		Engel grade		Test value	P-value	Sig.
		Grade 1	Grade 2			
		n=15	n=5			
Complications	No‎	13 (86.7%)	2 (40.0%)	4.356*	0.037	S
	Yes	2 (13.3%)	3 (60.0%)			
Types of complications	None	12 (80.0%)	3 (60.0%)	0.800*	0.371	NS
	Residual part of the lesion	0 (0.0%)	2 (40.0%)	6.667*	0.009	HS
	Behevioral changes	1 (6.7%)	0 (0.0%)	0.351*	0.553	NS
	None and with AEDs withdrawal the seizures recurrence so more	1 (6.7%)	0 (0.0%)	0.351*	0.553	NS
	Contralateral hemiparieses	1 (6.7%)	0 (0.0%)	0.351*	0.553	NS
Follow up duration (years)	Mean±SD	1.52±0.48	1.40±0.55	0.458•	0.653	NS
	Range	1–2	1–2			

In summary, this research highlights the extremely significant association between GBM and postoperative complications and the role of total resection of the lesion in achieving the best outcomes. Complications were strongly related to poor seizure control, in that two (40%) of the complicated group had Engel grade 1, whereas 13 (86.7%) of the non-complicated group did. In addition, left-sided lesion and male gender were predictors of poor Engel grade outcome. While there were no differing surgical methods among groups, trans-sylvian method predicted favorable seizure outcome in patients with Engel grade 2. These findings indicate the importance of tailored surgical methods and total resection of the lesion in order to decrease complications and increase long-term seizure control. Further studies with larger cohorts are needed to validate these results and identify other predictors of postoperative status.

## Discussion

Surgical intervention for epilepsy has been recognized as an extremely potent treatment for intractable TLE, particularly in cases where patients fail to achieve seizure control with AEDs. Though most patients with epilepsy respond to medication, approximately one-third of them do not respond. In drug-resistant focal-onset epilepsy, surgery, particularly anterior temporal lobectomy, has proven to be highly safe and effective in either eliminating seizures or significantly reducing their frequency. [10,11]. Despite such documented benefits, surgery remains underutilized; patients will spend years on several AEDs before a surgical referral is sought [12].

The complex anatomy of the MTL, which is intimately related to important structures such as the hippocampus and amygdala, presents unique challenges for surgical resection [13]. This complexity demands a tailored approach, which often involves adjunctive procedures like amygdalohippocampectomy for best seizure control [8]. Our own experience of 20 patients undergoing a resection of a medial temporal lesion with or without a subsequent amygdalohippocampectomy confirms growing evidence that not only does surgery improve the seizures but preserve essential cognitive functions.

The most frequent lesions in our series were gangliogliomas (n=4, 20%), then GBM (n=3, 15%) and pilocytic astrocytoma (n=3, 15%). This is consistent with previous reports, which suggest that low-grade tumors such as gangliogliomas are more frequently associated with drug-resistant epilepsy compared to high-grade lesions [5,14]. Interestingly, our review found no significant correlation between lesion type and Engel outcome-contrary to research like Giulioni (2013), which observed higher Engel Class I results in patients with low-grade tumors associated with epilepsy. This could be attributed to the small number of cases or variability in patient selection and surgery.

Our results also indicated that (n=15, 75%) of the cases were Engel grade I, whereas only (n=5 ,25%) were Engel grade II.

These findings closely resemble those from recent studies. For example, it is indicated that approximately 74% of the patients had achieved freedom from seizures post surgery. Of particular interest, subgroup analysis found that female patients and right-sided lesion patients had better outcomes with surgery, and a trans-sylvian approach was associated with poorer outcomes [15]. Although our study did not find a substantial relationship between demographic factors (e.g., age or seizure onset) and outcomes, these findings do suggest that patient-related factors and surgical approaches are worthy of further investigation.

Postoperative problems in five of our patients (25%) were residual lesion, change in behavior, return of seizures, and hemiparesis on the opposite side. Though these problems are modest and better than infection rates reported in other series, they do indicate that there should be careful surgical planning and technique [16]. Additionally, our findings concur with the recent studies, in which the long-term benefits of epilepsy surgery were emphasized, not only in seizure control but also on quality of life [17].

Overall, our study corroborates the data that early aggressive surgical intervention for drug-resistant TLE can produce significant changes in seizure control and quality of life. Underuse of epilepsy surgery is a persistent issue; as suggested by recent reviews, early referral for surgical evaluation may dramatically alter the course of the disease in the great majority of patients. Future studies to validate these observations will require larger sample sizes and longer follow-up duration [18,19].

There are some limitations to our study. The modest sample size (n=20) may influence the power of our conclusions, in the sense that it may not be representative of the larger population of drug-resistant epilepsy patients. Longer follow-up in the more complex cases may also have influenced outcomes. We also failed to report postoperative neuropsychological assessments. Furthermore, we did not control for variations in surgical technique or postoperative care, both of which impact outcome. Future larger prospective studies are required to validate our findings and identify other predictors.

## Conclusions

A medial temporal lobectomy, alone or in combination with amygdalohippocampectomy, serves as an effective surgical treatment for drug-resistant TLE. Most patients experience both a reduction in seizure frequency and severity while seizure freedom occurs in the majority after the procedure. These results support surgical management as an essential treatment option for medically refractory epilepsy and highlight the importance of identifying patients for early surgical evaluation. More studies with bigger sample sizes along with extended follow-up periods should be performed to improve patient selection criteria and surgical methods that enhance patient outcomes.

## References

[1] Adry RA, Meguins LC, Pereira CU, Silva Júnior SC, Araújo Filho GM, Marques LH (2018). Auras as a prognostic factor in anterior temporal lobe resections for mesial temporal sclerosis. Eur J Neurol.

[2] Fernández-Miranda JC, de Oliveira E, Rubino PA, Wen HT, Rhoton AL Jr (2010). Microvascular anatomy of the medial temporal region: part 1: its application to arteriovenous malformation surgery. Neurosurgery.

[3] Kucukyuruk B, Richardson RM, Wen HT, Fernandez-Miranda JC, Rhoton AL Jr (2012). Microsurgical anatomy of the temporal lobe and its implications on temporal lobe epilepsy surgery. Epilepsy Res Treat.

[4] Blümcke I, Pauli E, Clusmann H (2007). A new clinico-pathological classification system for mesial temporal sclerosis. Acta Neuropathol.

[5] Phi JH, Chung CK (2009). Brain tumors in the mesial temporal lobe: long-term oncological outcome. Neurosurg Focus.

[6] Soeder BM, Gleissner U, Urbach H, Clusmann H, Elger CE, Vincent A, Bien CG (2009). Causes, presentation and outcome of lesional adult onset mediotemporal lobe epilepsy. J Neurol Neurosurg Psychiatry.

[7] Ibrahim A, Rashad M (2022). Surgical management for drug-resistant epilepsy in medial temporal lobe lesions (Al-Azhar experience). Al-Azhar Int Med J.

[8] Uda T, Kunihiro N, Nakajo K, Kuki I, Fukuoka M, Ohata K (2018). Seizure freedom from temporal lobe epilepsy with mesial temporal lobe tumor by tumor removal alone without hippocampectomy despite remaining abnormal discharges on intraoperative electrocorticography: Report of two pediatric cases and reconsideration of the surgical strategy. Surg Neurol Int.

[9] Kanchanatawan B, Limothai C, Srikijvilaikul T, Maes M (2014). Clinical predictors of 2-year outcome of resective epilepsy surgery in adults with refractory epilepsy: a cohort study. BMJ Open.

[10] Wiebe S, Blume WT, Girvin JP, Eliasziw M (2001). A randomized, controlled trial of surgery for temporal-lobe epilepsy. N Engl J Med.

[11] Jobst BC, Cascino GD (2015). Resective epilepsy surgery for drug-resistant focal epilepsy: a review. JAMA.

[12] Wiebe S, Jetté N (2012). Epilepsy surgery utilization: who, when, where, and why?. Curr Opin Neurol.

[13] Goodman AM, Szaflarski JP (2021). Recent advances in neuroimaging of epilepsy. Neurotherapeutics.

[14] Su X, Chen HL, Wang ZY, Lan Q (2015). Relationship between tumour location and preoperative seizure incidence in patients with gliomas: a systematic review and meta-analysis. Epileptic Disord.

[15] Feys O, Goldman S, Lolli V (2023). Diagnostic and therapeutic approaches in refractory insular epilepsy. Epilepsia.

[16] Mathon B, Navarro V, Bielle F (2017). Complications after surgery for mesial temporal lobe epilepsy associated with hippocampal sclerosis. World Neurosurg.

[17] Giulioni M, Marucci G, Martinoni M (2013). Seizure outcome in surgically treated drug-resistant mesial temporal lobe epilepsy based on the recent histopathological classifications. J Neurosurg.

[18] Stavem K, Bjørnaes H, Langmoen IA (2008). Long-term seizures and quality of life after epilepsy surgery compared with matched controls. Neurosurgery.

[19] Lowe AJ, David E, Kilpatrick CJ, Matkovic Z, Cook MJ, Kaye A, O'Brien TJ (2004). Epilepsy surgery for pathologically proven hippocampal sclerosis provides long-term seizure control and improved quality of life. Epilepsia.

